# Navigating Virtual Learning Landscapes: Perspectives of Health Sciences Students in Saudi Arabia During the COVID-19 Pandemic

**DOI:** 10.7759/cureus.58504

**Published:** 2024-04-18

**Authors:** Jana H Alharbi, Fatma T Sherbini, Nouf A Alzahri, Mohamed E Ahmed, Raju S Kumar

**Affiliations:** 1 Department of Respiratory Therapy, College of Applied Medical Sciences, King Saud bin Abdulaziz University for Health Sciences & King Abdullah International Medical Research Center (KAIMRC), Jeddah, SAU; 2 Department of Basic Sciences, College of Science and Health Professions, King Saud bin Abdulaziz University for Health Sciences & King Abdullah International Medical Research Center (KAIMRC), Jeddah, SAU

**Keywords:** students, perception, mirror neurons, learning, body language

## Abstract

Introduction

In December 2019, COVID-19 originated in Wuhan, China, triggering a global pandemic. However, the Saudi Arabian Ministry of Education ensured the safe continuation of teaching and learning activities. Amid the pandemic, health sciences students were exposed to diverse learning opportunities.

Methods

This study seeks to explore their experiences with online teaching. Conducted as a descriptive cross-sectional study, it involved 397 health sciences students from three universities in the Makkah province who had encountered both traditional and virtual teaching methods.

Results

Most participants were female (71.1%), predominantly from Jeddah city (76.5%). The highest agreement scores were observed for student comprehension during online sessions (61.1%). A significant proportion (74.4%) found paying attention during online lectures easier than traditional ones. Blackboard emerged as the preferred educational platform for online teaching. Notably, there were no significant variations in students' perceptions of online teaching based on location, gender, or specialisation. Approximately 54.7% of students preferred watching their instructors through a webcam during online lectures.

Conclusion

Medical educators can leverage these findings to develop standardised teaching protocols and enhance the effectiveness of online education systems. The study underscores the importance of instructors using webcams during online teaching sessions, as it allows students to visually connect with their instructors, potentially improving the learning experience.

## Introduction

In December 2019, Wuhan, China, first reported COVID-19, an infectious disease caused by coronavirus. The World Health Organization (WHO) reported that individuals with this viral infection experienced mild to severe respiratory diseases that could call for specialized care. COVID-19 started to spread all around the globe quickly and mysteriously, and within a short period, it became a worldwide pandemic [1,2]. Due to this worldwide pandemic, governing bodies started to put restrictions on controlling the spread of the disease and started a lockdown that changed every aspect of human life [1].

The pandemic affected every sector of our lives, including the mode of education delivery. Consequently, a total shift to online learning was implemented, and schools and universities worldwide moved their education system away from the conventional instructor-delivered didactic lectures towards online teaching [3]. There was a paradigm shift in the teaching and learning process. Both teachers and students were forced to use various online platforms to continue their teaching and learning activities [4]. It was difficult for the educational sector to cope with the challenging changes [5]. Moreover, innovative tools and learning management systems for both teaching and evaluation have advanced, offering a usable alternative for educators and encouraging policymakers to implement the use of information technology during the quarantine days to cover coursework [6]. However, in higher education, online learning is becoming increasingly popular [1].

In Saudi Arabia, the Ministry of Education decided to continue the teaching and learning process safely and securely; consequently, they announced virtual teaching and online assessment strategies for all universities, including health sciences universities across the country, and this action was taken in adherence with the WHO’s social distancing protocol to avoid the spread of COVID-19 [1]. Virtual learning has many benefits, such as encouraging self-directed learning for students' curricula and upgrading and the possibility of recording online lectures, making it more adaptable for better understanding and student learning [7,8]. However, there are also reports suggesting the disadvantages, such as for students transitioning from traditional to online education, the sudden implementation of online teaching methods poses a considerable barrier [9]. Moreover, it was challenging for the students and teachers in various health sciences courses, especially medical sciences students, as it affected the laboratory and clinical teaching modalities in medical universities and hospitals during COVID-19 [10].

The COVID-19 pandemic affected medical education from different perspectives, such as students’ clinical exposure, laboratory experience, practising surgical procedures, and the absence of communication with patients [11]. Therefore, online teaching should not only focus on knowledge delivery but also consider student interaction with educational tools for getting progressive feedback. A study on nursing students revealed that blended learning positively impacted student learning [12]. Many physicians were worried about the possible lack of student interaction with the teachers during virtual learning, and their lack of attention could be an obstacle that would affect students’ attendance, enthusiasm for learning, and the development of their skills [13]. Because web cameras were turned off during online teaching sessions at many colleges, students could not view their instructors. They only hear their lecturers' voices, which could be hindering because one of the most common ways of communicating socially to provide and give valuable information to another individual is through their body language [14].

This study explores the virtual learning experiences and educational strategies of health science students from different universities across Saudi Arabia, particularly on gathering perspectives from students in the Makkah province.

The objectives of this study are to explore students' opinions on online teaching, compare it with traditional teaching methods, evaluate whether students comprehend the subjects taught, and determine their preferences for instructional approaches. This investigation aims to fill a significant void in the existing literature by addressing the gap in research among health science students from diverse university backgrounds.

## Materials and methods

This cross-sectional study examined health science students in three universities in Makkah Province, Saudi Arabia. Inclusion criteria encompassed students in health sciences programs who received traditional and virtual instruction from the respective universities: King Saud bin Abdulaziz University for Health Sciences, King Abdulaziz University, and Umm Al-Qura University. Exclusion criteria were applied to students lacking experience with virtual teaching. Utilising a convenience nonprobability sampling approach, participants were selected based on availability, accessibility, and willingness to engage in the study. The required sample size was determined using Raosoft® (Sample Size Calculator; Raosoft Inc., Seattle, USA), targeting a 5% margin of error and a 95% confidence level [15], resulting in an estimated sample size of n=385. However, the study enrolled 397 participants.

Validation of questionnaire

A self-administered validated questionnaire was used in the study (see Appendices). Four independent subject experts assessed the content validity of the questionnaire, while a team of medical education specialists evaluated the face validity. A pilot study was conducted on 40 participants. Cronbach’s alpha (α = 0.91) was calculated to determine the questionnaire's internal consistency. The participants who took part in the pilot study were not included in the original study. The questionnaire had four sections. The first section included the demographic details of the participants. The second section contained nine questions about Students' perception of online teaching. The third section consisted of eleven questions that compared online teaching with conventional (traditional) teaching methods. The fourth section had nine questions that assessed the students' perceptions of understanding the subjects taught and their choices of teaching-learning methods adopted by the teachers during virtual lectures. The students responded to the questionnaire on a five-point Likert scale: strongly agree, agree, neutral, disagree, and strongly disagree. The responses marked as agree or strongly agree were grouped as overall agreement to simplify the analysis. In contrast, those marked as disagreeing or strongly disagreeing were considered an indication of disagreement. This section also included three questions with closed-ended formats, offering respondents three predetermined options to choose from.

Ethical clearance

The study protocol, questionnaire, and consent forms were approved by the Institutional Review Board (IRB) with approval number SP21J-388-08

Data collection

A questionnaire survey was created using Google Forms (Google LLC, Menlo Park, USA) and distributed to students at three chosen universities in Makkah province through email and WhatsApp. All participants were fully informed about the survey's purpose and gave their voluntary consent to participate. Between September and October 2021, 397 students responded to the survey.

Data analysis

Data collected was entered into a Microsoft Excel spreadsheet (Microsoft Corporation, Redmond, USA) and processed and encoded for statistical analysis. The statistical software used was JMP 16.1 (SAS Institute, Cary, USA). For continuous variables, the data was presented as the mean and standard deviation (SD), while for categorical variables, the data was presented as frequencies and percentages. ANOVA and an independent t-test were used to explore associations between students' characteristics and responses, with results having P values less than or equal to 0.05 considered significant.

## Results

Results related to the descriptive statistics

The study gathered responses from 397 (n = 397) completed survey questionnaires focusing on health sciences students in Makkah Province, Saudi Arabia. Most participants (76.5%) were from Jeddah. Among the respondents, 282 (71.1%) were females. The survey included a significant representation from King Saud bin Abdulaziz University for Health Sciences, Jeddah (n = 219, 55.3%), and King Abdulaziz University, Jeddah (n = 88, 22.3%). The participants were predominantly fourth-year students (153; 38.7%), followed by third-year students (114; 28.9%).

Results related to inferential statistics

Table 1 indicates that the highest agreement scores among students' perceptions during virtual lectures were 61.1%. Conversely, at 30.2%, the least preferred option was recording online lectures. Regarding the time allocated for online sessions, 51.6% of participants agreed it was sufficient for their learning.

**Table 1:** Students’ perception of online teaching (n=397)

Item	Mean	% Of agreement
Are you satisfied with the online teaching initiatives at your university?	2.75	54.4
Online teaching helps build your knowledge.	2.97	59.4
My level of understanding during online teaching sessions is very good.	3.05	61.1
I could clearly understand the course learning objectives during online teaching sessions.	2.76	55.2
There is a lack of common learning goals during the online teaching-learning.	2.68	53.6
Do you feel that you are less committed during the online learning process	2.29	45.8
You often feel a lack of clarity in understanding the concepts in the subjects that are taught during online lectures	2.59	51.6
Do you feel that the time duration allotted for the online lectures is enough to fulfil your understanding of the course	2.59	51.6
It is more helpful if the online lectures are recorded	1.51	30.2
Overall	2.58	51.53

In Table 2, 74.4% of respondents claimed they could pay more attention to lecturers during online lectures than traditional lectures. When considering attending laboratory sessions in person versus online lab sessions during the COVID-19 pandemic, only 34.2% believed in-person attendance would be more beneficial.

**Table 2:** Students' perceptions regarding comparing online teaching with conventional (traditional) teaching. (n=397)

Item	Mean	% of agreement
Online teaching saves your time and effort compared to traditional teaching.	2.25	45.0
Online teaching is better in building teamwork and collaborative learning between students.	3.42	68.4
The online teaching-learning process is more fun-filled compared to traditional teaching.	3.46	69.2
Online and traditional teaching has the same quality regarding your ability to understand.	3.39	67.8
Paying attention to the teachers is easier during online lectures compared to traditional lectures	3.72	74.4
You feel that physical attendance during laboratory sessions in the college is more beneficial than online laboratory sessions.	1.71	34.2
You feel online Learning is more distracting than traditional classroom learning	2.26	45.2
You find yourself more productive during the online learning process than in-classroom learning.	3.08	61.6
You are more enthusiastic about attending online lectures than physically attending university lectures.	3.39	67.8
You feel your grades/scores in the subjects have significantly improved during online teaching compared to traditional lectures.	2.91	58.2
You feel your teachers can better explain and clarify your doubts during online lectures than physical lectures.	3.25	65
Overall	2.99	59.7

**Figure 1:**
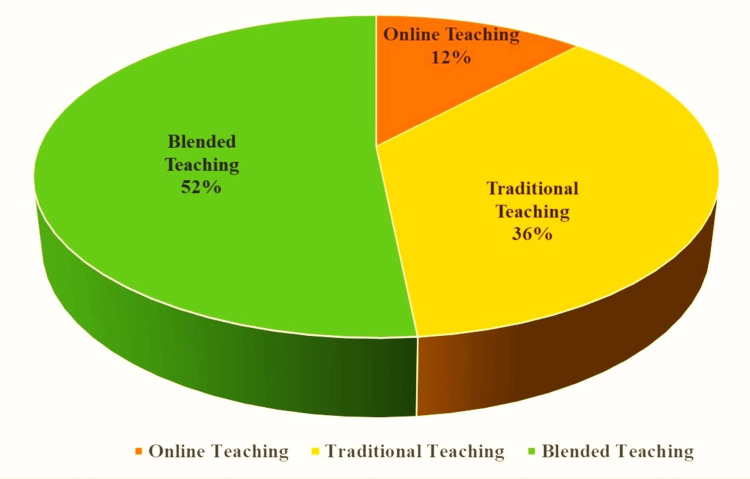
Teaching strategy preferred by the students.

Table 3 presents means and percentages of agreement related to students' perceptions of understanding subjects and preferences for teaching strategies during virtual lectures. The overall agreement rate was 48.09%. Notably, 49.8% of students agreed on the availability of teachers to clarify doubts after working hours. About 54.7% of students preferred watching instructors through the webcam during virtual classes. Most students (74.4%) favoured Blackboard (Anthology, Reston, USA)as their educational platform for online learning. Additionally, 52% preferred blended lectures (combining online and traditional teaching) over online or traditional lectures alone, as represented in (Figure 1).

**Table 3:** Students' perspectives regarding the subjects taught and instructors' teaching methods during virtual lectures. (n=397)

Item	Mean	% of agreement
During online teaching, the teachers always encourage students to ask them questions.	2.22	44.4
During online teaching, you feel that your teachers always try to involve you in the lectures by asking questions.	2.48	49.6
Teachers are always available for the students to clarify doubts outside their teaching hours.	2.49	49.8
You could pay more attention during online lectures if your teachers ask you more questions.	2.51	48.2
The revision sessions your teachers give during online sessions on previous lectures help you understand subjects better.	2.09	41.8
You prefer seeing your instructors through their web camera while they deliver online lectures.	2.73	54.7
Overall	2.42	48.09

Table 4 highlights substantial variation in overall perceptions of online teaching among health sciences students of different academic years. Sixth-year students expressed greater satisfaction and agreement with online teaching than in other years. However, students' location, gender, and subject speciality showed no significant differences in their perceptions of online instruction.

**Table 4:** Student's perception in comparison between online teaching and conventional (traditional) teaching by basic characteristics of students (n=397) *Significant at 5% level

Variable	Perception mean	P-value
City		0.384
Jeddah	2.56	
Makkah	2.62	
Gender		0.211
Female	2.59	
Male	2.52	
Specialty		0.516
Medicine	2.53	
Applied Medical Sciences	2.61	
Nursing	2.63	
Others	2.62	
Year of Study		0.011*
First-year	2.74	
Second year	2.65	
Third year	2.61	
Fourth year	2.50	
Fifth Year	2.51	
Sixth year	3.35	
Internship	2.58	

The results in Table 5 summarise students' overall perceptions of online teaching based on surveyed characteristics. Notably, there is a significant gender difference, with female students expressing higher satisfaction with online teaching than their male counterparts. Additionally, students in the sixth year significantly show more satisfaction and agreement with online teaching. However, students' location and subject speciality did not significantly differ in their perceptions of online teaching.

**Table 5:** Students' perception of online teaching by basic characteristics of students (n=397) *Significant at 5% level

Variables	Perception mean	P-value
City		0.169
Jeddah	2.96	
Makkah	3.07	
Gender		0.007*
Female	3.04	
Male	2.83	
Specialty		0.117
Medicine	2.93	
Applied Medical Sciences	2.98	
Nursing	3.02	
Others	3.31	
Year of Study		0.011*
First year	3.00	
Second year	3.17	
Third year	3.06	
Fourth year	2.82	
Fifth Year	3.05	
Sixth year	3.52	
Internship	2.97	

## Discussion

During the COVID-19 pandemic, online learning has become integral to the education sector. Its popularity has grown significantly in higher education as it provides an effective way to teach and impart knowledge. The pandemic has had a significant impact on the global education industry. In this research, we examined the opinions of health sciences students regarding the teaching and learning methods employed in various courses amid the COVID-19 pandemic in the Makkah province of Saudi Arabia. The individuals involved in our study reported enhanced comprehension and increased focus during online lectures. This could be attributed to their potential greater ease within their home environment, devoid of disruptive elements that could impede their concentration. These results are consistent with a prior investigation involving Polish medical students who preferred to continue their online education within the comfort of their residences [11]. A small percentage of students believed attending physical laboratory sessions in college would be more efficient because it would improve their hands-on experience with skills and concepts. Most students, however, chose to persist with their online laboratory sessions, potentially driven by apprehensions about contracting COVID-19. This concurs with the findings of a meta-analysis study, which revealed that college students harbour a moderate level of fear associated with the pandemic [16].

According to a study, Zoom emerged as the most widely used online learning tool, followed by WhatsApp and Google Classroom. Microsoft Teams, Edmodo, Skype, and Google Meet were employed moderately [17]. Nevertheless, our results indicated a predominant preference among medical students for Blackboard as their primary educational platform, followed by Microsoft Teams and Zoom during online instruction. It has been asserted that open-source tools like Blackboard, Zoom, Google Meet, Microsoft Teams, and WebEx see broader usage in online medical education than proprietary software. Blackboard is notably prevalent in numerous public universities in Saudi Arabia, establishing it as a familiar tool for students.

A significant association between gender and attitudes toward online education was observed in our study. Among the participants, comprising 71% female students, a higher proportion held a positive view of online learning than their male counterparts. This aligns with the research by Liu X et al., where female students (68.7%) demonstrated superior performance in online learning, reinforcing the consistency of our findings [18]. As per research conducted in Malaysia, women tend to exhibit higher levels of emotional self-regulation compared to men. [19]. Studies suggest that women generally demonstrate elevated levels of emotional intelligence compared to men [20]. Our research findings imply that female students held a more positive view of online learning than their male counterparts, thus substantiating these hypotheses.

51.6% of participants in our study preferred blended lectures over traditional or online lectures. This finding supports a meta-analysis indicating that students hold favourable attitudes towards blended learning [21]. Students participating in online education may experience decreased involvement and an increased likelihood of dropping out [21]. Students' learning styles and readiness differ. It has been reported that blended learning is a valuable strategy for managing students' readiness, motivation, and learning styles [22].

According to our survey, 30.2% of students found having online lectures recorded for future reference helpful. However, most students disagreed with recording sessions and preferred to watch live online lectures. This contradicts a previous report, which found that students preferred recorded lectures because they allowed them to study at their convenience [23]. A recent study suggests that recorded lectures may negatively impact some students' attendance and learning habits [24]. Our study found that 54.7% of students preferred seeing their teachers on a web camera during online sessions Table 3.

Correct use of body language techniques can enhance student learning [25]. Albert Mehrabian was the first to introduce the 7/38/55 communication rule [25]. According to this rule, verbal communication accounts for only 7% of human interaction. The remaining 93% is conveyed through non-verbal cues such as tone and inflection of voice (38%) and body language (55%), including hand gestures, posture, and facial expressions. We believe that when instructors opt not to turn on their web camera during lectures, it could lessen the lecture's impact as students cannot see the instructor's hand gestures and facial expressions while they deliver the online lecture. According to Mehrabian's rule, verbal communication accounts for only 5% of human communication. Functional magnetic resonance imaging (fMRI) has shown that communicative hand gestures activate the mirror neuron system in the human brain [26]. Studies have reported that mirror neurons can aid in the process of learning [27]. We propose that when students in online classes observe their instructors' communicative hand gestures and body language, the activation of mirror neurons may enhance their learning process. However, additional research is necessary to confirm this hypothesis. In our study, sixth-year students expressed excellent satisfaction with virtual teaching. This aligns with a study from Saudi Arabia, where senior health sciences students also reported positive satisfaction with virtual learning [28]. This finding could be due to a multifactorial reason. We hypothesize that senior students in medical and health sciences might have already adapted to hectic academic schedules, assignments, and examinations, while junior students might be in the beginning stages of coping with academic stress. Research has shown that stress levels decrease as students’ progress from their first to final year [29,30]. Based on this hypothesis, we believe that sixth-year students might be less stressed in our study than their juniors, which could be one reason for higher satisfaction towards virtual teaching. However, further research is needed to prove this hypothesis. 

Limitations and recommendations

This study may be limited by its relatively small sample size of 397 participants, primarily comprising females, which could potentially constrain its representativeness. A larger and more diverse sample from multiple regions and universities is suggested to enhance the study's robustness. Longitudinal studies are imperative for comprehending the lasting impacts of online education. Thus, including a broader and more varied sample of health sciences students would bolster the study's external validity. Furthermore, it is recommended that faculty training workshops be organized to refine online teaching techniques and incorporate virtual tools to elevate the calibre of online education.

## Conclusions

In conclusion, this study sheds light on the perspectives of health sciences students towards online education amidst the COVID-19 pandemic in the Makkah province of Saudi Arabia. The findings reveal a general acceptance and satisfaction with online teaching and learning despite specific challenges and preferences identified by the participants. Notably, there is a consensus among students regarding the benefits of online lectures in enhancing comprehension and attention. The study also highlights the importance of instructors' non-verbal communication, particularly body language, in facilitating effective online teaching. The preference for seeing instructors through web cameras underscores the significance of visual cues in student engagement and learning.

Overall, the findings contribute to the ongoing discourse on online education and underscore the need for continuous improvement and adaptation of teaching methods to meet students' evolving needs in the digital age. Additionally, recommendations for faculty training workshops aim to enhance the quality of online education and ensure effective and engaging learning experiences for students in health sciences programs.

## Appendices

Questionnaire

I. Basic demographics of participants

1.      In which city within the Western region of Saudi Arabia do you live?

2.     What is your gender identity?

3.     At which university are you currently enrolled?

4.     Please specify the academic year you are currently in.

Students.

5.     What is your field of specialization?

II. Student perception of online teaching

All responses were recorded using a 5-point Likert scale, ranging from "Strongly agree" to "Strongly disagree."

1.     Are you satisfied with the online teaching initiatives at your university?

2.     Online teaching helps build your knowledge.

3.     My level of understanding during online teaching sessions is very good.

4.     I could clearly understand the course learning objectives during online teaching sessions.

5.     There is a lack of common learning goals during the online teaching-learning.

6.     You feel that you are less committed during the online learning process.

7.     You often feel a lack of clarity in understanding the concepts in the subjects that are taught during online lectures.

8.     You feel that the time allotted for the online lectures is enough to fulfil your understanding of the course.

9.     It is more helpful if the online lectures are recorded.

III. Students' perceptions regarding comparing online teaching with conventional (traditional) teaching.

All responses were recorded using a 5-point Likert scale, ranging from "Strongly agree" to "Strongly disagree."

1.     Online teaching saves your time and effort compared to traditional teaching.

2.     Online teaching is better in building teamwork and collaborative learning between students.

3.     The online teaching-learning process is more fun-filled compared to traditional teaching.

4.     Online and traditional teaching have the same quality regarding your understanding ability.

5.     Paying attention to the teachers is easier during online lectures compared to traditional lectures.

6.     You feel that physical attendance during laboratory sessions in the college is more beneficial than online laboratory sessions.

7.     You feel online Learning is more distracting than traditional classroom learning.

8.     You find yourself more productive during the online learning process than in-classroom learning.

9.     You are more enthusiastic about attending online lectures than physically attending university lectures.

10.  You feel your grades/scores in the subjects have significantly improved during online teaching compared to traditional lectures.

11.  You feel your teachers can better explain and clarify your doubts during online lectures than physical lectures.

IV. Students' perspectives regarding the subjects taught and instructors' teaching methods during virtual lectures.

Except for the first, third and ninth questions, the responses to other questions were recorded using a 5-point Likert scale, ranging from "Strongly agree" to "Strongly disagree."

1.     What teaching approach employed by your instructors do you favour for enhancing the learning experience?

A.    Online Teaching

B.    Conventional (Traditional) teaching

C.    Blended lectures (Includes both online and traditional teaching)

2.     During online teaching, the teachers always encourage students to ask them questions.

3.     Which educational platform do you prefer for your online lectures?

A.    Blackboard

B.    Microsoft teams

C.    Zoom

D.    Others

4.     During online teaching, you feel that your teachers always try to involve you in the lectures by asking questions.

5.     Teachers are always available for the students to clarify doubts outside their teaching hours.

6.     You could pay more attention during online lectures if your teachers ask you more questions.

7.     The revision sessions your teachers give during online sessions on previous lectures help you understand subjects better.

8.     You prefer seeing your instructors through their web camera while they deliver online lectures.

9.      Which of the following teaching strategies do you feel can help you find yourself with more free time?

A.    Online Teaching

B.    Conventional (Traditional) teaching

C.    Blended lectures (Includes both online and traditional teaching)
